# Thermophilic Co-Digestion of the Organic Fraction of Municipal Solid Wastes—The Influence of Food Industry Wastes Addition on Biogas Production in Full-Scale Operation

**DOI:** 10.3390/molecules23123146

**Published:** 2018-11-30

**Authors:** Przemysław Seruga, Małgorzata Krzywonos, Marta Wilk

**Affiliations:** Department of Bioprocess Engineering, Wrocław University of Economics, Komandorska 118/120, 53-345 Wrocław, Poland; przemyslaw.seruga@ue.wroc.pl (P.S.); marta.wilk@ue.wroc.pl (M.W.)

**Keywords:** methane, anaerobic digestion, restaurant waste, stillage, chocolate waste

## Abstract

Anaerobic digestion (AD) has been used widely as a form of energy recovery by biogas production from the organic fraction of municipal solid wastes (OFMSW). The aim of this study was to evaluate the effect of the introduction of co-substrates (restaurant wastes, corn whole stillage, effluents from the cleaning of chocolate transportation tanks) on the thermophilic anaerobic digestion process of the mechanically separated organic fraction of municipal solid wastes in a full-scale mechanical-biological treatment (MBT) plant. Based on the results, it can be seen that co-digestion might bring benefits and process efficiency improvement, compared to mono-substrate digestion. The 15% addition of effluents from the cleaning of chocolate transportation tanks resulted in an increase in biogas yield by 31.6%, followed by a 68.5 kWh electricity production possibility. The introduction of 10% corn stillage as the feedstock resulted in a biogas yield increase by 27.0%. The 5% addition of restaurant wastes contributed to a biogas yield increase by 21.8%. The introduction of additional raw materials, in fixed proportions in relation to the basic substrate, increases biogas yield compared to substrates with a lower content of organic matter. In regard to substrates with high organic loads, such as restaurant waste, it allows them to be digested. Therefore, determining the proportion of different feedstocks to achieve the highest efficiency with stability is necessary.

## 1. Introduction

Anaerobic digestion (AD) is a naturally occurring process in which organic matter is converted into a biogas (a combination of methane and carbon dioxide) and a digestate (semi-solid residue), by microorganisms in the absence of oxygen [1,2,3,4]. However, the process can be engineered and optimized in dedicated plants to maximize the biogas production.

The anaerobic digestion is accomplished by a series of biochemical transformations, which can be separated into four steps: hydrolysis, acidogenesis, acetogenesis, and methanogenesis. The process can be conditioned by various operating factors. Among them, the most impacting process performance factors are feed rate, solid content, temperature, and retention time [1,2,4,5,6].

Conventional municipal solid wastes (MSW) management is based on disposal to land-fill, where AD occurs naturally. A system of wells and pipes collects the biogas which can be burnt in Combined Heat and Power (CHP) units to produce heat and electricity or burnt directly in a flare. Although land-filling is a simple and low-cost method of wastes utilization, it is associated with water and air pollution. The ideal actions are to avoid, reduce, reuse, recycle, recover, and treat to minimize disposal and focus on a circular economy [7,8,9,10,11,12]. Processing the biogas by AD before land-filling leads to a reduction of methane discharge to the atmosphere and decreased greenhouse gas emissions, smells, and sanitary disadvantages of landfills.

During the last few years, anaerobic digestion of the organic fraction of municipal solid wastes (OFMSW) has been used widely as a form of energy recovery via biogas and many researchers, businesses, and government agencies are working to improve the process [3]. It can be seen that AD is the most promising and sustainable process for the treatment of organic wastes and it is an environmentally friendly method for MSW management [6,13,14,15].

In Europe, about 90% of the full-scale plants for AD rely on a one-stage system and this is divided into wet and dry digestion [12,16]. Since the start of this century, the number of thermophilic AD plants has grown significantly in Europe [17] due to the positive results in thermophilic anaerobic treatment reported in the literature and the main advantages of thermophilic conditions in comparison to mesophilic ones, such as less time required for higher loads treatment, increase in biogas production, and better hygiene of digestate due to higher destruction of pathogenic microorganisms.

Besides operating under thermophilic conditions, the addition of co-substrates might also increase the biogas production of a single OFMSW digestion process. Due to this possibility, some biogas plants are searching for wastes with higher biogas potentials. On the other side, high organic matter content single substrate digestion, such as restaurant wastes or corn whole stillage, might lead to operational problems [18,19]. For example, high protein content results in a high nitrogen content after hydrolysis, which affects the concentration of ammonia or ammonium ions in the digester and might inhibit the process. The stability of the digestion process can also be influenced by the accumulation of volatile fatty acids and long chain fatty acids, due to a high organic load of the substrate [15]. Thus, co-digestion should be considered as a possibility for controlling the AD stability process and maximizing biogas production.

The balance of nutrients, C/N ratio, minerals and metals content, and increased buffering system capacity impacts the digestion process. Therefore, co-digestion enhances process stability, organic matter biodegradation, and biogas and methane yields [20,21,22,23]. A number of studies on the anaerobic digestion of corn stillage, food wastes, or OFMSW as a single substrate can be found in the literature [2,7,24,25,26,27,28]. Also, research regarding the OFMSW co-digestion of food waste, sewage sludge, and other feedstocks has been reported [4,11,12,25,29,30,31,32,33]. However, information on co-digestion of the OFMSW with corn stillage and effluents from the cleaning of chocolate transportation tanks is scarce in the literature.

The aim of this study was to evaluate the effect of using co-substrates in the thermophilic anaerobic digestion process of the mechanically separated organic fraction of municipal solid wastes in a full-scale mechanical-biological treatment (MBT) plant. The OFMSW co-digestion with three different feedstocks: restaurant wastes, corn whole stillage, effluents from the cleaning of chocolate transportation tanks, considering three addition ratios (5, 10, and 15% OFMSW input) was evaluated in terms of the biogas yield and the stability of the process.

## 2. Results and Discussion

The full-scale pilot plant was evaluated according to single substrate AD with the OFMSW and compared to the AD with addition of different types of co-substrates (restaurant wastes (RW), corn whole stillage (CS), effluents from the cleaning of chocolate transportation tanks (ETC)).

**Figure 1:**
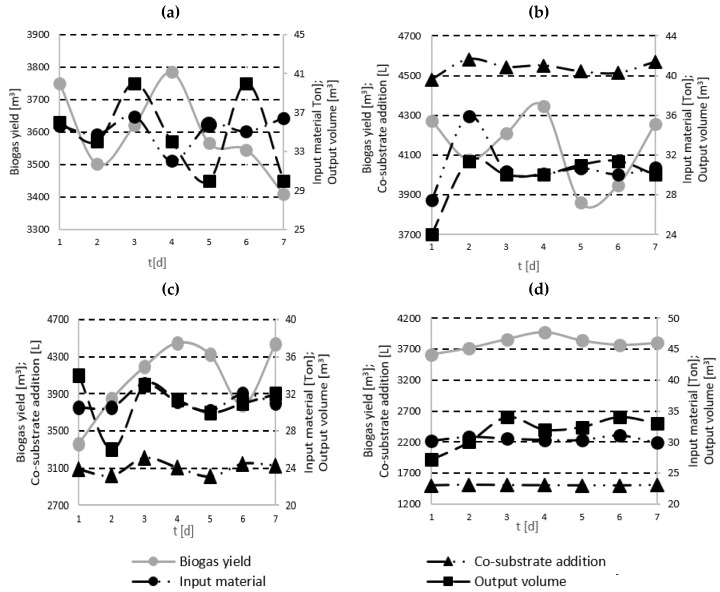
Parameter changes during 7-day anaerobic digestion runs: (**a**) OFMSW, (**b**)OFMSW + 15% effluents from tanks cleaning, (**c**) OFMSW + 10% corn stillage, (**d**) OFMSW + 5% restaurant wastes.

The anaerobic digestion of the single OFMSW carried out in the MBT plant was a stable process. The historical exploitation trends are similar to the single AD run performed in this study (Figure 1a). The biogas yield was around 100 L/Kg of input material at similar daily and weekly levels. The feeding load fluctuated around 35 Ton per day (Figure 1a). The filling of the digester was kept at the level of approximately 1135 m^3^ (Figure 1a). The concentration of acetic acid was around 1.0 g/Kg and the concentration of propionic acid was around 160 mg/Kg. The contents of both forms of the butyric acid were maintained at the same level at the beginning and end of the process. The acidity/alkalinity ratio (index R) was 0.4. The recorded hydraulic retention time (HRT) was 32.2 days.

Anaerobic digestion brings greater benefits compared to aerobic composting, mainly due to the possibility of energy recovery from wastes [34]. However, the non-uniformity and variability of fraction content and also the inert fraction’s (glass, stones, etc.) content negatively affect OFMSW digestion process efficiency [2]. The biogas yield (about 100 L/Kg) and methane content (52–53%) noted in this study were similar to results obtained in other facilities in Poland and laboratory-determined biogas potentials [35]. However, due to the variety of OFMSW, comparison of the obtained results with those of facilities operating in Europe or elsewhere in the world is difficult. Campuzano and González-Martínez [7] reviewed results in the composition of OFMSW and methane yield for 43 cities in 22 countries around the world. The obtained methane yield ranged from 61 L/Kg up to 580 L/Kg, with an average value of 415 ± 138 L CH_4_/Kg. In Italy, the average methane yield was 400 L/Kg, while in Denmark it was 499 L [7].

The pH value is considered to be the anaerobic digestion process stability factor [36,37]. Normally, it should be between 7.2 and 7.8. However, it might be affected by temperature, sampling, and might be characteristic for the facility. A pH-value below 7 results in a decrease in the activity of microorganisms responsible for volatile fatty acid decomposition, and thus a drop in biogas production. pH-value decreases are usually caused by organic overloading of the digester [36,37]. The increase in alkalinity (pH values above 8.0) impacts the NH_3_ and NH_4_^+^ dissociation balance. High pH values and elevated temperature conditions favour the accumulation of NH_3_ (aq), which can pass through the microbial membrane, thus inhibiting microbial activity [36,37]. However, mainly due to the digester buffer capacity, pH changes are usually only detectable when the process is already unstable. Therefore, it is found that the determination of the low-fatty acids (acetic, propionic, butyric, and valeric) concentrations, with their ratios, provides the most reliable information concerning digestion stability [36,37].

**Figure 2:**
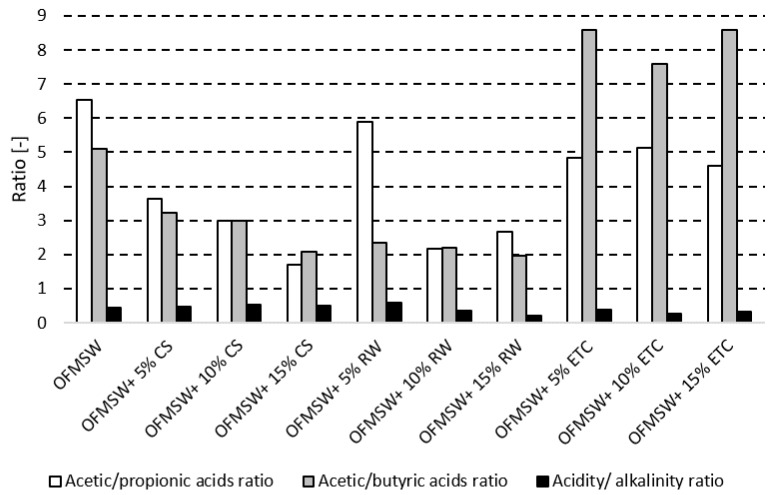
Fatty acids’ concentrations and acidity/alkalinity ratios for single substrate AD with the OFMSW and the AD with addition of different types of co-substrates: corn stillage (CS), restaurant wastes (RW), and effluents from the cleaning of chocolate transportation tanks (ETC).

The single OFMSW anaerobic digestion process was stable. The acetic acid content was noted at 1.0 g/Kg, with the concentration of propionic acid between 155 and 170 mg/Kg and their ratio between 5.9 and 6.5 (Figure 2). The R index was 0.4 (Figure 2). However, the expected AD process benefits, because of a low production of electricity and heat, were not achieved. 

After 5% addition of the effluents from the cleaning of chocolate transportation tanks, the AD process was stable. The daily input of the OFMSW was 30 Ton and liquid feedstock was 1510 L. The biogas yield was around 109 L/Kg of the input material at similar daily and weekly levels. An increase in the acetic acid concentration from 0.97 g/Kg to 1.13 g/Kg was reported. Nevertheless, the concentration of propionic acid decreased from about 400 to about 230 mg/Kg. A four-fold increase in the butyric acid content was observed. The values of valeric acid content were stable during the process. A 10% addition of the effluents (introduction of 3100 L of liquid feedstock with 31 Ton OFMSW to the digester per day) did not affect process stability. Biogas yield of 115 L/Kg was noted. At the end of the co-digestion run, a 30% increase in the acetic acid concentration was noted. It was accompanied by a rise in the concentration of propionic acid (from about 80 to about 200 mg/Kg) and butyric acid (from 3 to 11 mg/Kg). The valeric acid content remained at a similar level during the process. With a 15% addition of the effluents, the co-digestion process remained stable. The daily input of the OFMSW was 30 Ton and liquid feedstock was 4500 L (Figure 1b). The biogas yield was around 135 L/Kg of the input material at similar daily and weekly levels (Figure 1b). At the end of the co-digestion run, increases in the following fatty acids concentrations were observed: acetic acid (by 40%), propionic acid (from about 80 to 200 mg/Kg), and butyric acid (from 3 to 11 mg/Kg). The valeric acid content remained at a similar level during the process. The increase of addition of the effluents resulted in a rise in fatty acids concentration and in the biogas yield, compared to the single OFMSW anaerobic digestion process, without process disruption.

The 5% addition of the corn stillage resulted in a biogas yield of 110 L/Kg. The amount of introduced OFMSW was at the level of 30.7 Ton/d with 1550 L liquid co-substrate. The concentrations of acetic, propionic, butyric, and valeric fatty acids were the same at the beginning and end of the run. With a 10% addition of stillage, the biogas yield was around 130 L/Kg. The daily input of the OFMSW was 31 Ton with 3100 L liquid feedstock (Figure 1c). At the end of the co-digestion run, increases in the following fatty acid concentrations were observed: acetic acid (from 0.73 to 1.57 g/Kg), propionic acid (from 161 to 523 mg/Kg), butyric acid (from 106 to 522 mg/Kg), and valeric acid (from 49 to 140 mg/Kg). Addition of 15% stillage (introduction of 4500 L of liquid feedstock with 30.5 Ton OFMSW to the digester per day) caused a significant increase in the acid content. The acetic acid concentration increased from 1.01 to 1.66 g/Kg. A growth in the propionic acid content (from 233 to 980 mg/Kg) was also noted. The butyric acid and valeric acid concentrations increased by 750 and over 20 mg/Kg, respectively. Compared to previous runs, acid contents were at the highest levels. Following a 10% addition of corn stillage, the observed acid concentrations were doubled. Regarding co-digestion with 15% addition of the effluents, acetic and propionic acid contents rose by about 60% and 320%, respectively. The concentrations of other acids were also many times higher.

**Figure 3:**
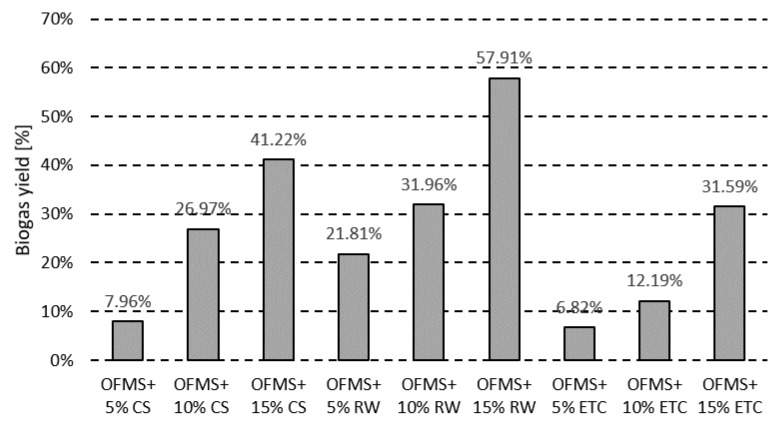
Biogas yield increment in co-digestion with addition of different types of co-substrates: corn stillage (CS), restaurant wastes (RW), and effluents from the cleaning of chocolate transportation tanks (ETC).

The 5% additions of effluents from tank cleaning and corn stillage resulted in an almost 7–8% rise in biogas yield (Figure 3), corresponding to a 14.3–16.8 kWh production increase (Table 1), without process interruption. Increasing the addition of the effluents to 15%, resulted in a biogas yield increase by 31.6% up to 134.6 L/Kg (Figure 3) with an almost 68.5 kWh production increase (Table 1). The acetic acid content oscillated around 1.0 g/Kg, while propionic acid was below 250 mg/Kg, with the ratio of these acids above 4.6 (Figure 2). No risk of disturbances in the digestion process was noted [36,37].

Introduction of 10% and 15% additions of corn stillage resulted in biogas yields of 130 and 144 L/Kg, respectively, corresponding to 58.5 and 89.3 kWh yields (Table 1). 

An increase in acid concentrations was noted. The acetic to propionic acid ratio exceeded 2.99 and 1.69, respectively, in the 10% and 15% co-digestion runs (Figure 2). It was found that AD process stability values were exceeded [36,37] and addition of corn stillage ought to be limited to a maximum of 10%. Eskicioglu et al. [38] compared biogas and methane yields for anaerobic digestion under meso- and thermophilic conditions. For organic loads of 6348, 12,696, 25,696, and 50,786 mg, COD/L biogas yields of 788 ± 24, 715 ± 14, 720 ± 11, and 691 ± 5 mL/g for mesophilic conditions and 1041 ± 22, 881 ± 16, 772 ± 37, and 635 ± 8 mL/g for thermophilic conditions were noted, respectively [38]. The average biogas yield exceeded 96 ± 19 L per stillage litre in thermophilic conditions and 88 ± 8 L of biogas per stillage litre [38]. However, with regard to whole corn stillage (12.4% dry matter content, organic load of 254 g COD/L) under thermophilic continuous digestion, a significant increase in the propionic acid content was noted after the 30th day. The rise in the concentration of propionic acid to about 3.0 g/L and an increase in acetic to propionic acid ratio to 2.6 resulted in a biogas production slump [38]. Complementing the technical process of a biogas plant with an ethanol production distillery brings an improvement in its energy balance. The savings in the energy consumption of the process were determined as 52.21% and 57.06%, respectively, for thermophilic and mesophilic conditions, regarding distillery production of 3.78 × 108 litres of ethanol annually [39,40]. However, it should be noted that this is a very expensive investment.

In the next stage of the study, the impact of restaurant waste addition was examined. With a 5% addition of the restaurant waste, the AD process remained stable. The daily input of the OFMSW was at the level of 30.5 Ton with 1500 L of liquid feedstock (Figure 1d). The biogas yield was around 124 L/Kg of the input. At the end of the run, an almost 32% increase in the acetic acid content was noted. Also, the concentrations of the remaining acids increased by over 70 mg/Kg, over 370 mg/Kg, and about 44 mg/Kg for propionic, butyric, and valeric acids, respectively, and were at the highest level among the examined 5% additions of the analyzed feedstocks. With a 10% addition of food waste, the biogas yield was around 135 L/Kg. The daily input of the OFMSW was 31.5 Ton with 3050 L of liquid feedstock. It was revealed that addition of restaurant waste resulted in a significant rise in fatty acid content. The acetic acid concentration increased from 0.98 to 1.44 g/Kg. The propionic acid concentration increased from 311 to 660 mg/Kg. Also, the contents of butyric acid and valeric acid increased from 69 to 649 mg/Kg, and 29 to 40 mg/Kg, respectively. The acid concentration increases were much higher than the values obtained with 10% addition of the other considered feedstocks. With 15% addition of restaurant waste, the feeding load was 30 Ton of OFMSW per day with 4500 L co-substrate. A 161.5 L/Kg biogas yield was noted. Significant fatty acid concentration increases were observed, compared to initial values by over 1.42, 0.68, 1.32, and 0.12 g/Kg for acetic, propionic, butyric, and valeric acids, respectively. 

When restaurant waste co-digestion was performed, increments in low fatty acid content were observed. The acid ratios, as well as acidity to alkalinity ratio, decreased with increasing restaurant waste addition (Figure 2). Although a significant biogas yield increment was noted (almost 58% regarding a 15% supplementation (Figure 3)), process stability values were exceeded [36,37] and addition of restaurant waste ought to be limited to a maximum of 5%.

Pavi et al. [15] verified the biogas yield obtained from co-digestion of the biodegradable fraction from mixed municipal wastes with fruit and vegetable wastes mixed in different ratios (1:0, 1:3, and 0:1). A constant level of volatile solids was maintained for each mixture. The highest yields of biogas (29.04 mL/g/day) and methane (23.32 mL/g/day) were noted with the ratio of 1:3. In comparison to the organic fraction fermentation, the increment in biogas yield exceeded 141% [15]. In our research, increases in co-substrate addition resulted in increased fatty acid concentration and biogas yield. Furthermore, the highest biogas yield of 161.5 L/Kg was noted with 15% food waste addition.

Brown and Li [41] conducted co-digestion of green wastes from parks with food wastes coming from supermarkets in a 1 L bioreactor at 36 °C. The impact of 10% and 20% food waste addition on the process was verified. The supplementation resulted in an almost 1.5 and 2.8-fold increase in the daily maximum methane yields, respectively, for 10 and 20% additions. However, regarding the co-digestion with 20% addition of food waste, between the 10th and 20th process days, a biogas production slump was observed, resulting from volatile fatty acids accumulation [41]. The impact of restaurant waste addition on the sludge from wastewater treatment plants digestion was verified by Dai et al. [42]. Regarding single AD, 2.1 L production of methane per day was obtained. Increasing the addition of food wastes resulted in a rise in methane yield (5.62 L) for a mass ratio of 2:3. Due to restaurant waste digestion, the methane yield exceeded 8.22 L/d. However, disturbances of the process were noted and the trial was unsuccessful. Co-digestion affects process stability [42]. Bodík [43] verified the impact of the restaurant wastes addition on the sewage sludge digestion process. As a result of the introduced organic load, decreasing pH-value, and low biogas yield, the trial was unsuccessful [43]. The biogas yield was found to be related to the reactor’s organic load. After exceeding certain values, the process breaks down, which resulted in a biogas production slump [42,43]. The addition of high organic-matter-content waste resulted in increased fatty acid concentrations and biogas yield. However, after exceeding certain values, overloading of the reactor was observed.

Co-digestion affected the concentrations of fatty acids, alkalinity, acidity, and biogas yields (Figure 2). The addition of effluents from the cleaning of chocolate transportation tanks did not impact process stability. The acetic to propionic acid ratio (R-value) remained above 4.6, with the acidity to alkalinity ratio ranging from 0.28 to 0.35 (Figure 2). For restaurant waste co-digestion, decreased acetic to propionic acid ratio with the increment in the amount of feedstock was noted, as well as an R-value decrease (Figure 2). Similar effects were noted for the corn stillage co-digestion. The decrease in the acetic to propionic acid ratio along with an increase in the amount of feedstock was noted. Meanwhile, the R-value remained at 0.48–0.53 (Figure 2). A rise in biogas yield was observed in all co-digestion runs (Figure 3), ranging from almost 7% in the case of the effluent addition, to almost 58% from restaurant waste co-digestion. 

For anaerobic digestion of OFMSW and all co-digestion runs, the pH value was maintained within 8.0–8.2. Dry matter content and ammonium ion concentration remained stable at 32–35% and 2.8–3.2 g NH_4_-N /Kg, respectively. Methane content in the biogas was also stable at a level of 52–53%. These values correspond with important stability limits for parameters characterizing the biogas process [37] and exploitation of historic trends.

Hydraulic retention time was stable and was kept at a similar level to reduce its impact on fatty acid concentration in co-digestion runs. The lowest (31 days) was obtained for co-digestion with 15% addition of effluents from the cleaning of chocolate transportation tanks. The highest HRT (35.6 days) was for 5% addition of restaurant waste.

The biogas yield determines the electricity production potential, which is the main profit from anaerobic digestion. Even an increase of a few percent in the biogas yield results in higher electricity production. For co-digestion with 5% addition of effluents from the cleaning of chocolate transportation tanks or corn stillage, increases from 6.9 to 8.1 L/Kg of biogas yield were noted, which correspond to 14.3–16.8 kWh of energy produced (Table 1). The highest rise in the electricity production yield (125.5 kWh) was noted in OFMSW with 15% addition of restaurant waste (Table 1). Yearly electricity yield ranged from 57.4 to 502.0 MWh (Table 1).

Based on the results of this study, as well as those of other researchers, it can be seen that co-digestion might provide benefits and process efficiency improvements, compared to monosubstrate digestion. The introduction of additional raw material in fixed proportions to the basic substrate affects the biogas yield due to substrates having a lower content of organic matter. This allows substrates with high organic loads to be digested. Food wastes, due to their high biogas potential, are good organic substrates for anaerobic digestion. However, process disturbances were noted. Therefore, determining the proportions of feedstocks that achieve the highest efficiency with stability is necessary. Also, benefits from electricity production should be considered as an advantage of co-digestion (Table 1). Considering 40% CHP unit efficiency, a 10 L/Kg biogas yield leads to a 21.2 kWh electricity yield, corresponding to 84.8 MWh yearly production. With a 15% addition of effluents from chocolate transportation tanks, a 68.5 kWh electricity yield was observed, which can produce an additional 273.9 MWh per year. 

## 3. Materials and Methods

### 3.1. Organic Fraction of Municipal Solid Wastes (OFMSW)

The research was performed at the MBT plant located in the Lower Silesia Region, Poland. The OFMSW was mechanically separated by screening (drum screen 60 mm), glass, stones, and ceramics ballistic separation, and ferric and non-ferric metals separations. The composition of the OFMSW is presented in Table 2.

### 3.2. Feedstock Used for Co-Digestion

The corn stillage was collected from distillery plants in Poland. The restaurant wastes were collected from restaurants and kitchens in the Lower Silesia Region of Poland. The effluents from the cleaning of chocolate transportation tanks were collected from a chocolate factory in Poland. The characteristics of the feedstocks used in the research can be found in Table 3.

### 3.3. Process Conditions

The anaerobic digestion processes were carried out in 1500 m^3^ full-scale Kompogas^®^ reactors. A daily load of 30 Ton of OFMSW was provided. Due to co-digestion 5, 10, and 15% of OFMSW input additions to each feed cycle were introduced. The temperature was maintained at 54 °C. The agitation speed was set at 0.45 rpm in cycles of 600 s operation with a 60 s break in alternating directions. The operating volume in the digester (about 1100 m^3^) was determined by setting the extraction pump operation time. The biogas stream was measured using a flowmeter. Its composition (content of methane, hydrogen sulfide, and oxygen) was determined using a biogas analyzer (ADOS Biogas 401). Process parameters were archived daily by the central control system. Parameter changes during 7-day anaerobic digestion runs were measured. Samples from the digester were collected every week. Between runs, the two-week acclimation period of each treatment was established. Average values are reported. 

### 3.4. Analytical Methods

The pH value, dry matter content, acidity, alkalinity, content of fatty acids (acetic, propionic, butyric, and valeric), and COD were determined. 

The content of fatty acids was determined by gas chromatography (Varian GC 450) with an FID detector (H_2_: 30 mL/min, air: 300 mL/min, He: 30 mL/min). Helium (constant flow through the 1mL/min column) was used as the carrier gas, 1:30 split.

The acidity and alkalinity were determined by pH-metric titration, according to standard methods [44]. The acidity/alkalinity ratio (index R) was also determined.

The suspended solids (SS) and dry organic mass were determined according to standard methods [44]. 

The chemical oxygen demand (COD) was established spectrophotometrically using Lange cuvette tests.

### 3.5. Calculation Methods

The weekly average biogas yield was calculated using the formula: Y = (V_av_/M_av_), where: Y is a biogas yield [m^3^/Ton], V_av_ is a biogas daily volume average over the course of a week [m^3^] and M_av_ is a daily feeding amount average over the course of a week (Ton).

The biogas yield increment for co-digestion was calculated using the formula: ΔY_n_ = [(Y_n_ − Y_0_)/Y_0_] × 100%, where ΔYn is a biogas yield increment [%], Y_0_ is a weekly average biogas yield obtained from the OFMSW digestion (m^3^/Ton) and Y_n_ is a weekly average biogas yield obtained from co-digestion (m^3^/Ton).

## 4. Conclusions

Based on these results, it can be observed that co-digestion of OFMSW with organic-matter-rich wastes is an appropriate method for process efficiency improvement. However, it should be limited. The 15% addition of effluents from the cleaning of chocolate transportation tanks resulted in an increase in biogas yield by 31.6%, to 134.6 L/Kg, corresponding to a 68.5 kWh electricity production possibility. Introduction of 10% corn stillage as the feedstock resulted in a biogas yield increase of 27.0%, to 129.9 L/Kg, corresponding to a 58.5 kWh electricity production possibility. The 5% addition of restaurant waste contributed to a biogas yield of 124.5 L/Kg (21.8% increase), corresponding to a 47.1 kWh electricity production possibility. 

## Figures and Tables

**Table 1:** Electricity production efficiency.

Input Material	Biogas yield (m^3^/Ton)	Biogas yield increment (m^3^/Ton)	Yearly biogas yield increment (m^3^)	Methane content (%)	Electricity yield (kWh)	Yearly electricity yield (MWh)
OFMSW	102.3	-	-	52	-	-
OFMSW + 5% corn stillage	110.4	8.1	32,400	52	16.85	67.40
OFMSW + 10% corn stillage	129.9	27.6	110,400	53	58.51	234.05
OFMSW + 15% corn stillage	144.4	42.1	168,400	53	89.25	357.01
OFMSW + 5% restaurants waste	124.5	22.2	88,800	53	47.06	188.26
OFMSW + 10% restaurants waste	135.0	32.7	130,800	53	69.32	277.30
OFMSW + 15% restaurants waste	161.5	59.2	236,800	53	125.50	502.02
OFMSW + 5% effluents from tanks cleaning	109.2	6.9	27,600	52	14.35	57.41
OFMSW + 10% effluents from tanks cleaning	114.7	12.4	49,600	53	26.29	105.15
OFMSW + 15% effluents from tanks cleaning	134.6	32.3	129,200	53	68.48	273.90

* quantities calculated based on MBT plant capacity: Yearly biogas yield increment based on Biogas yield increment and 4000 Ton MBT yearly limit of food waste quantity; Electricity yield according to formula: Biogas yield increment × Methane content × Electrical production possibility (10 kWh/m^3^CH_4_) × CHP unit efficiency (40%); Yearly electricity yield according to formula: Yearly biogas yield increment × Methane content × Electrical production possibility (10 kWh/m^3^CH_4_) × CHP unit efficiency (40%).

**Table 2:** The composition of the OFMSW.

Fraction	Mass Share
Organic	54.1 ± 3.8%
Wood	10.1 ± 1.2%
Paper	1.6 ± 0.5%
Plastics	4.9 ± 0.5%
Glass	3.1 ± 1.1%
Inert waste	3.5 ± 0.8%
Textiles	1.0 ± 0.1%
Metals	0.1 ± 0.1%
Hazardous	0.1 ± 0.1%
Tetrapack	1.1 ± 0.2%
Others	0.3 ± 0.1%
Fine fraction 0–15 mm	20.1 ± 4.2%

**Table 3:** Feedstocks characterization.

Parameter	Corn Whole Stillage	Restaurant Wastes	Effluents from Tanks Cleaning
pH	4.1	5.9	6.3
SS (%)	10.9	22.3	18.8
COD (g/L)	120.1	230.0	105.0

## Founding

This research received no external funding.
